# The AudioGene Translational Dashboard for Diagnosing Autosomal Dominant Nonsyndromic Hearing Loss: Phenotypic Data Visualization and Analysis Study

**DOI:** 10.2196/85212

**Published:** 2026-04-14

**Authors:** Benjamin DeSollar, Nathan Schaefer, Daniel Walls, Amanda M Odell, Kevin T A Booth, Hela Azaiez, Michael Schnieders, Richard J H Smith, Terry Braun, Thomas Casavant

**Affiliations:** 1 Department of Electrical and Computer Engineering, University of Iowa, 103 South Capitol Street, Room 5316, Iowa City, IA, United States, 1 319-335-5953; 2 Department of Otolaryngology, Head and Neck Surgery, University of Iowa, Iowa, IA, United States; 3 Department of Medical and Molecular Genetics, Indiana University, Indianapolis, IN, United States; 4 Department of Otolaryngology—Head and Neck Surgery, School of Medicine, Indiana University, Indianapolis, IN, United States; 5 Department of Biochemistry and Molecular Biology, University of Iowa, Iowa City, IA, United States; 6 Department of Molecular Physiology and Biophysics, University of Iowa, Iowa City, IA, United States; 7 Department of Biomedical Engineering, University of Iowa, Iowa City, IA, United States

**Keywords:** autosomal dominant nonsyndromic hearing loss, machine learning, explainable artificial intelligence, clinical decision support systems, genotype-phenotype correlation, audiometry, genetic testing

## Abstract

**Background:**

Autosomal dominant nonsyndromic hearing loss (ADNSHL) is highly heterogeneous, with more than 64 genes implicated in its etiology. This complexity limits the diagnostic power of clinical examinations and audiometry alone, while existing computational approaches have achieved only moderate accuracy and often lack interpretability. As precision medicine increasingly emphasizes genotype-phenotype correlations, there is a recognized need for diagnostic tools that provide clinicians with transparent, interpretable outputs.

**Objective:**

This study aimed to develop and evaluate the AudioGene Translational Dashboard, an interpretable clinical informatics tool that integrates machine learning models and interactive visualizations to enhance genotype-phenotype correlations and support diagnostic decision-making in ADNSHL.

**Methods:**

We developed the AudioGene Translational Dashboard, integrating 2 machine learning models (AudioGene version 4 and AudioGene version 9.1) with 6 interactive visualization tools. AudioGene version 4 uses a multi-instance support vector machine classifier for patients with multiple audiograms, while AudioGene version 9.1 combines adaptive boosting, k-nearest neighbors, random forest models, and logistic regression for patients with a single audiogram. Visualizations include audiometric profile plots, audioprofile surfaces, clustering analyses, and data distribution charts designed to facilitate clinical interpretation.

**Results:**

The AudioGene Translational Dashboard was developed to address the “70/30” phenomenon, indicating a 74% likelihood that the causative gene is among the top 3 predicted genes, thereby providing clinicians with a clear confidence indicator (“green flag”) or a caution alert (“red flag”) during diagnosis. While this level of performance is well suited for hypothesis generation, the remaining uncertainty underscores the need for interpretive context in clinical decision-making. Visualization tools enhanced clinicians’ ability to interpret and correlate phenotypic data with predicted genetic outcomes, improving diagnostic confidence and interpretability.

**Conclusions:**

The AudioGene Translational Dashboard advances clinical informatics in genetic diagnosis of ADNSHL by integrating explainable artificial intelligence with interactive visualizations, enhancing clinical interpretability and diagnostic accuracy. This approach facilitates informed clinical decision-making, highlights the translational potential of genotype-phenotype computational models, and supports precision medicine in hearing loss diagnostics. Future enhancements will target improving class balance and incorporating additional user-customizable features to further optimize clinical applicability.

## Introduction

### Background

Autosomal dominant nonsyndromic hearing loss (ADNSHL) presents a significant genetic diagnostic challenge due to its underlying heterogeneity—more than 64 genes are implicated in its etiology [1]. Because of this complex genetic landscape, computational tools designed to correlate audiogram profiles (commonly called audioprofiles) with specific genes have achieved only moderate success [2,3]. One such tool, which we developed approximately 15 years ago, is AudioGene. AudioGene uses numerous machine learning (ML) approaches to improve diagnostic precision [2,4,5]. These approaches include semisupervised support vector machines (SVMs), ensemble models, and hyper-tuning methods. However, challenges such as data imbalance and class sparsity continue to restrict the accuracy of these models.

Precision medicine harnesses information about the genome of an individual, environment, and lifestyle to guide medical care. With heterogeneous disorders such as ADNSHL, genetic variant interpretation can be challenging, complicating the diagnostic process and impacting patient care. Computational tools may improve the precision and reliability of genetic assessments by capitalizing on genotype-phenotype associations [6,7].

Current diagnostic methods for ADNSHL largely rely on clinical examination and audiometry, which do not provide sufficient resolution for the complex genetic landscape of ADNSHL [2,4]. However, with the availability of ML and artificial intelligence–driven approaches, there has been a shift toward integrating computational and visualization tools with genetic diagnostics to improve accuracy and predictive power [8,9].

To address these challenges, we have developed the AudioGene Translational Dashboard with the goal of enhancing both the accuracy and interpretability of genetic predictions. A feature of the AudioGene Translational Dashboard is the “70/30” phenomenon: by integrating the results from both models on a training dataset comprising 3189 audiograms from 1445 patients, we observed that the correct disease-causing gene was predicted within the top 3 predictions 74% of the time, with incorrect predictions accounting for the remaining 26%, hence “70/30.” This observation signals to health care providers when they can have confidence in the top predictions, serving as a “green flag” or “red flag” in the diagnostic process. Having a true positive rate of 70% is beneficial from a research perspective; however, for a diagnostic tool, the remaining 30% represents some risk that necessitates additional interpretative context. By providing this context, the AudioGene Translational Dashboard enables health care providers to weigh their confidence in the predictions, supporting more informed diagnostic decisions.

The AudioGene Translational Dashboard was introduced into the AudioGene toolset to increase transparency into the “black box” underlying the models by providing explainable artificial intelligence (XAI) to enhance model interpretability and utility in clinical settings, in line with trends in precision medicine that emphasize the importance of genotype-phenotype associations in improving diagnostic outcomes [7,10].

### Related Works

Early attempts to map audiometric phenotypes to their underlying genotypes were spearheaded by AudioGene version 4 (AG4), a semisupervised multi-instance SVM that treats the collection of audiograms for a single patient as a “bag” and ranks loci according to pair-wise–coupled probability estimates [2,11]. Building on this foundation, AudioGene version 9.1 (AG9.1) introduced selective intraensemble data partitioning: training examples are first divided by gene-specific data volume, patient age, and audiogram shape, then modeled with a committee of k-nearest neighbor (KNN), adaptive boosting, and random forest subclassifiers, whose outputs are fused by logistic regression. AG9.1 offers a top-3 accuracy of 77.8%, with a precision of 0.51 and a recall of 0.56, at the cost of introducing a more complex model. We report top-3 accuracy rather than top-1 accuracy because, in the context of gene prioritization for validation sequencing, the cost of excluding the true causative gene is higher than the cost of evaluating a small number of candidate genes. In addition, the top-3 threshold represents a practical trade-off between high confidence in predictions and an acceptable loss of significance when selecting genes for sequence-based validation [4,5,12,13]. Both frameworks have improved locus-ranking accuracy for the 23 well-curated ADNSHL genes that account for roughly three-quarters of cases in populations of European ancestry [14]. Nonetheless, their predictions can still be difficult to interpret when class imbalance, sparse age coverage, or atypical audiogram morphologies are present.

Complementary to algorithmic advances, domain-specific visualization has been welcomed as a potentially beneficial tool for clinical use. Audioprofile surfaces (APS) plot 3D trajectories of frequency-specific threshold shift over time, revealing gene-characteristic progression patterns that are not obvious in 2D audiograms [15]. Circle-based genome views (eg, Circos [version 0.69-10; Krzywinski, Canada’s Michael Smith Genome Sciences Center] enable high-density comparison of structural variation or copy number events [16], while integrative genome browsers such as Integrative Genomics Viewer (version 2.19.7; UC San Diego and Broad Institute of MIT and Harvard) allow rapid inspection of read evidence at candidate loci [17]. More recent health care dashboards use fuzzy logic overlays and interactive filtering to expose outliers or low-confidence regions directly to end users [18]. Despite these advances, few systems combine genotype-prediction engines with audiogram-aware visual contexts; therefore, clinicians must cross-reference separate tools, a workflow that can erode trust in algorithmic suggestions and slow decision-making [19]. These limitations reflect shortcomings in how models communicate their reasoning and how results are presented to end users.

Accordingly, the literature reveals two unmet needs:

1. Model transparency—while ensemble and semisupervised approaches improve predictive accuracy, they do not inherently communicate *why* a particular gene is ranked highly, especially when training data are imbalanced or noisy.
2. Unified, clinician-friendly interfaces—existing genomic viewers excel at sequence-level detail but lack phenotype-specific visualization; conversely, stand-alone audiogram tools rarely link observed hearing profiles back to the underlying variant evidence.

The AudioGene Translational Dashboard addresses these gaps by (1) merging the complementary strengths of AG4 and AG9.1 and (2) embedding 6 interactive visual modules—APS, 2D audioprofiles, uniform manifold approximation and projection (UMAP) cluster projections, gene count bar charts, region-of-origin pie charts, and age distribution plots—around the model output. This hybrid XAI-driven design supports clinicians in validating or questioning the algorithm’s “70/30” confidence observation and thus advances the state of practice in ADNSHL diagnostics.

## Methods

### Tool Overview

The AudioGene Translational Dashboard integrates 2 ML models to increase diagnostic accuracy for ADNSHL:

- AG4—it is a multi-instance classifier designed for patients with multiple audiograms that uses a semisupervised SVM and ranks loci based on modified SVM probability outputs [4]. It was developed using the Waikato Environment for Knowledge Analysis (version 3.7.2; WekaIO Inc) platform [11].
- AG9.1—it is a single-instance classifier for patients with only 1 audiogram, developed using the scikit-learn library in Python. It comprises multiple submodels: 3 KNNs, 6 adaptive boosting models, 2 random forest models, and a logistic regression module for combining outputs [2,5,8].

**Figure 1.**
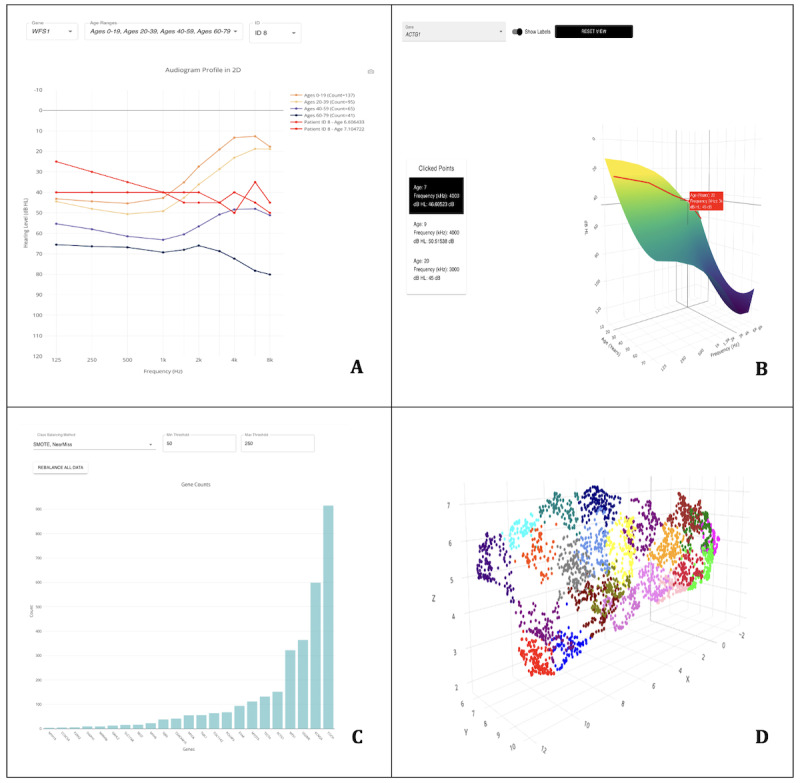
Visualization components of the AudioGene Translational Dashboard. (A) Audioprofile for the selected gene (*WFS1*), displaying data from all age ranges along with patient data; (B) audioprofile surface view for the selected gene (*ACTG1*); (C) count bar chart showing the counts of each gene in the training data; and (D) 3D uniform manifold approximation and projection of genetic case data used in the AudioGene Translational Dashboard.(each point represents a classified genetic case, and the color coding corresponds to 1 of the 23 unique clusters identified through different genetic diagnoses).

**Figure 2.**
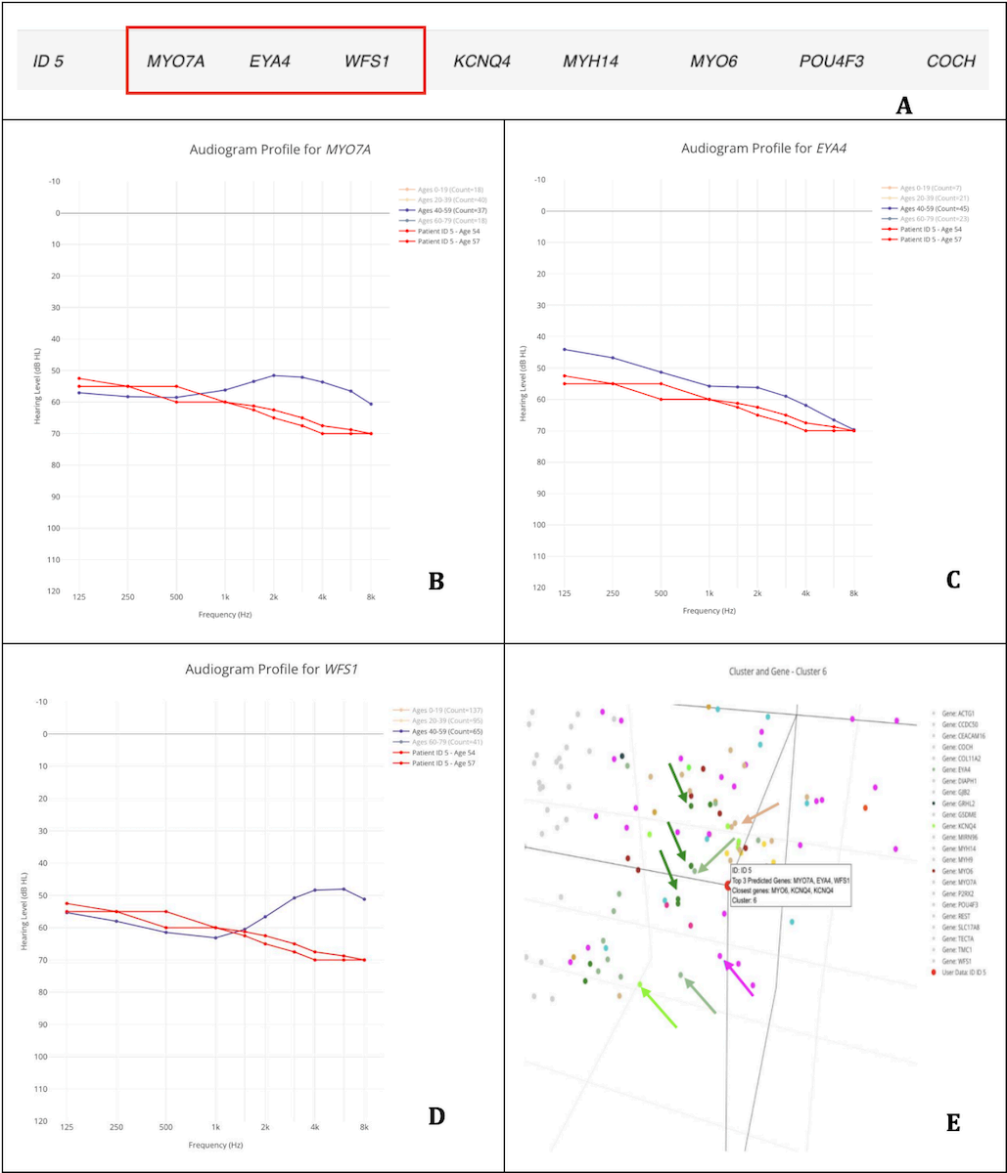
Application of the AudioGene Translational Dashboard for patient-level gene prediction and visualization. (A) Predictions for patient (ID 5), highlighting the top 3 genes associated with the audiological characteristics observed; (B) audioprofile of MYO7A with the patient’s (ID 5) audiograms in red, taken at the ages of 54 and 57 years; (C) audioprofile of EYA4 with the patient’s (ID 5) audiograms in red, taken at 54 and 57 years of age; (D) audioprofile of WFS1 with the patient’s (ID 5) audiograms in red, taken at the ages of 54 and 57 years; and (E) 3D plot of audiograms in the training set reduced to 3 dimensions for visualization, with genes in cluster 6 colored (genes not in cluster are light gray; patient [ID 5] is the red dot hovered over by the displayed label; and the green arrows point to MYO7A [green dots], the pale green arrows point to EYA4 [pale green dots], the pale yellow arrow points to WFS1 [pale yellow dots], the pink arrow points to COCH [pink dots], and the light green arrow points to KCNQ4 [light green dots]).

The AudioGene Translational Dashboard interface provides six distinct visualization tools for health care providers and researchers to interactively assess genetic data and model predictions in real time: (1) audioprofile, a 2D plot displaying the average hearing loss (in dB) over 10 frequencies (125 Hz to 8000 Hz) for each age group, allowing comparison with a patient’s hearing loss over time (Figure 1A); (2) APS, a 3D surface plot depicting gene-specific hearing loss progression over time across frequencies, illustrating age-related changes in decibel loss (Figure 1B) [20]; (3) a region-of-origin pie chart, which displays the geographic origin distribution (eg, Dutch, German, and Chinese) for audiograms associated with each gene; (4) a count bar chart, which illustrates the audiogram count for each gene, highlighting class imbalance challenges (Figure 1C); (5) spatial analysis and clustering, which shows the cluster position of each gene and a 3D plot of audiograms (clusters are created using the k-means algorithm to partition data into 23 gene-specific groups [15]; the 3D plot compresses 11 features [age plus 10 frequencies] into 3 dimensions using the UMAP method (Figure 1D) [21]); and (2) 6) an age distribution scatter plot, which shows the age distribution for each gene within the training dataset, providing context for the predictive model outputs.

The first 3 visualization tools were developed to compare patient-specific data to average thresholds for each of the 23 ADNSHL-associated genes. This comparison allows patient audiograms to be contextualized for each gene. The audioprofile visualization shows how a patient’s audiogram compares to the expected audiograms associated with each gene.

The APS adds time as the third axis to provide a 3D rendering of gene-specific audiometric thresholds over time. Audiometric thresholds are represented as a 3D plane, depicting the expected dB loss over time (in years) at each frequency, thereby enabling comparisons between a patient’s hearing thresholds and gene-specific expectations.

The spatial analysis and clustering tool uses a bar chart to show the distribution of each prediction among 23 different clusters. These clusters are created using k-means clustering [15]. Additionally, a 3D plot visualizes the audiograms within our data that have a confirmed genetic diagnosis, compressing 11 features into 3 dimensions using UMAP [21]. This feature allows users to interact with the bar chart, highlighting corresponding clusters in the 3D plot (Figure 1). Users can compare their patient’s audiogram, represented by a large red dot, with others in the cluster, facilitating the identification of similar audiograms and associated genetic diagnoses.

By using these 3 visualizations, we aim to either enhance or reduce confidence in the predictions. For example, if the model ranks the *COCH* gene second among the top 3 predictions, health care providers can analyze the APS and spatial analysis tools to determine the degree of correlation with patterns typically associated with the *COCH* gene, potentially increasing confidence in that diagnosis.

The last 3 visualization tools provide context for the data in the AudioGene dataset. The count bar chart highlights significant class imbalance, showcasing the challenge the model encounters in predicting smaller classes due to underrepresentation. The region-of-origin pie chart and age distribution scatter plot provide additional context about data distribution, allowing health care providers to understand model limitations and adjust diagnostic strategies accordingly.

The AudioGene Translational Dashboard integrates into the workflow of a clinician as a secondary validation layer. For example, within our clinical workflow, clinicians, genetic counselors, and bioinformaticians review the results of a clinical genetic test, including patient history, family structure, audiograms, and identified variants in hearing loss genes. This team can then inspect a patient’s audiometric data relative to the landscape of audiometric data across all genes and patients, considering variance within a gene, rarity or abundance of cases within a cluster, and distance to genetically validated cases.

### System Design

The system was designed using the SERN (SQL, Express.js, React.js, and Node.js) stack, which uses a client-server architecture where computationally intensive tasks are performed by the server, deployed in a Docker (version 28.5.1; Docker Inc) container [19,22]. The client is supported by React (version 18.2.0; React Foundation), a JavaScript library facilitating user interactions [23]. Data preprocessing used linear interpolation and extrapolation for missing values. The same methods for handling missing values were applied in the ML models [2,5,8].

The *Pandas* library in Python was used for data manipulation and analysis [8], and visualization libraries such as Plotly were used to create interactive graphs and plots [24].

For more information, please refer to the master’s thesis by DeSollar [3] and the GitLab repository.

### Ethical Considerations

This study was reviewed and approved by the University of Iowa Institutional Review Board (199701065). The institutional review board granted a waiver of informed consent under US federal regulation 45 CFR 46.116(f) (also known as the “Common Rule”), because, although audiograms were originally collected in clinical and research settings, the dataset used for this study was fully deidentified prior to analysis [25]. All procedures adhered to the ethical standards of the institutional and national research committees and to the 1964 Declaration of Helsinki and its later amendments. This paper does not contain any individual’s data in any form, including individual details, images, or videos. No compensation was provided to participants, as this study involved secondary analysis of a fully deidentified existing dataset and no participants were directly recruited or enrolled.

## Results

### Introduction to AudioGene Translational Dashboard

The AudioGene Translational Dashboard combines advanced ML models with several visualization tools to create a platform that facilitates the prioritization of ADNSHL-associated genes in genetic testing results. Using patient data, the AudioGene Translational Dashboard generates gene rankings and enables auditory scientists and health care providers to explore these predictions interactively through various visualization tools. Gene ranking in phenotype-genotype associations can aid in the interpretation of complex genetic data, thereby providing greater context and confidence in diagnostic decisions [5,15].

The “70/30” phenomenon serves as an indicator for health care providers, providing them with the necessary context through visualizations to assess the reliability of the predictions. When the top 3 predictions include the correct gene, health care providers can have greater confidence in proceeding with targeted genetic testing.

### Case Studies and Clinical Implications

Several case studies have been carried out to demonstrate the effectiveness of the AudioGene Translational Dashboard in the diagnosis of specific genetic types of ADNSHL [3].

#### Case 1: *MYO7A* Gene—Patient 1 (ID 5)

In this case study, we analyzed the results from a patient diagnosed with *MYO7A*-related hearing loss. Our dataset included 2 audiograms, which were predicted by AG4 to be associated with *MYO7A*-related, *EYA4*-related, or *WFS1*-related hearing loss (Figure 2A).

Examining these predictions relative to the patient’s audiograms, the following observations can be made: (1) *MYO7A’s* audioprofile is similar in the low-to-mid frequencies but diverges in the high frequencies (Figure 2B); (2) *EYA4* displays a comparable shape, but the patient’s thresholds are consistently lower than typical values (Figure 2C); and (3) *WFS1* shares some similarities in the low-to-mid frequencies but diverges in the high frequencies, as observed with *MYO7A* (Figure 2D).

The audioprofiles for the 3 genes all show moderate to moderately severe hearing loss thresholds, with either close similarity (<5 dB) at several frequencies or similar shapes. Therefore, we can conclude that the correct gene is likely captured within the top 3 predictions.

#### Case 2: *MYO6* Gene—Patient 2 (ID 2)

**Figure 3.**
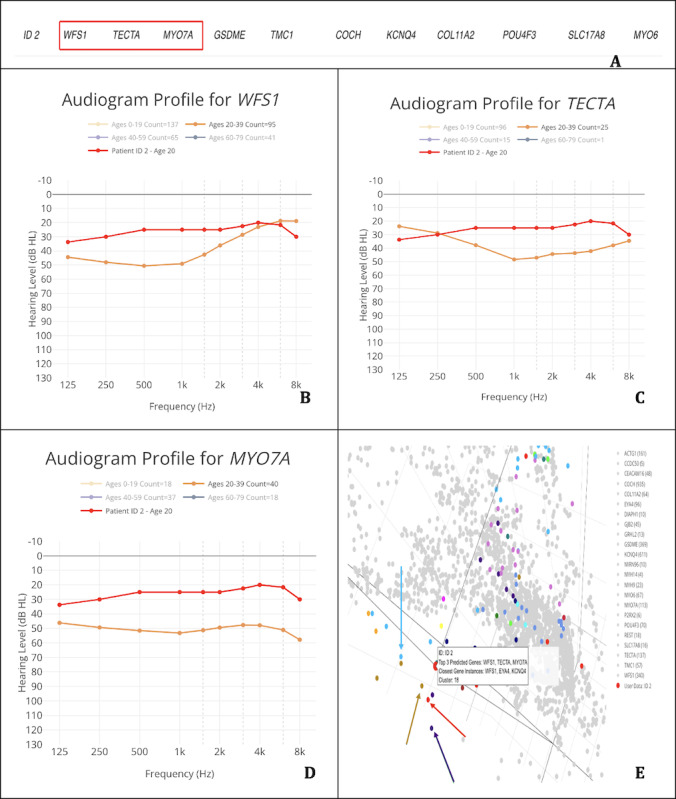
Application of the AudioGene Translational Dashboard for patient-level gene prediction, audioprofile comparison, and cluster-based visualization. (A) Predictions for the patient (ID 2), with the top 3 genes being WFS1, TECTA, and MYO7A; (B) audioprofile of WFS1 with the patient’s (ID 2) audiograms in red, taken at 20 years of age; (C) audioprofile of TECTA with the patient’s (ID 2) audiograms in red, taken at the age of 20 years; (D) audioprofile of MYO7A with the patient’s (ID 2) audiograms in red, taken at the age of 20 years; and (E) 3D plot of audiograms in the training set reduced to 3 dimensions for visualization, with genes in cluster 18 colored (genes not in the cluster are light gray; patient [ID 2] is the red dot hovered over by the displayed label; and the light blue arrow points to KCNQ4 [light blue dots], the red arrow points to WFS1 [red dots], the purple arrow points to POU4F3 [purple dots], and the brown arrow points to GSDME [brown dots]).

In our second case, we explored the results from a patient (ID 2) previously diagnosed with *MYO6*-related hearing loss. Our dataset contains only 1 audiogram, with gene predictions shown in Figure 3A by AG9.1.

None of the top 3 candidate genes (*WFS1*, *TECTA*, or *MYO7A*) display an audioprofile that closely aligns with the patient’s thresholds (Figure 3B–D). This mismatch strongly suggests that the true causative gene (*MYO6*) does not appear among the model’s top 3 predictions for this patient.

From the clustering interface, we observe that the genes closest to the patient’s audiogram by the KNN metric (*WFS1*, *EYA4*, and *KCNQ4*) also fail to match the patient’s observed audioprofile in any convincing way. Furthermore, *KCNQ4*, *GSDME*, and *POU4F3*, which are noted to have multiple data points near the patient’s cluster, likewise show audioprofiles inconsistent with the patient’s hearing loss. These factors combine to produce inconclusive predictions in this case. When we then integrated these data with the genetic data—which identified no genetic variants in *WFS1*, *TECTA*, or *MYO7A* and confirmed a known variant in *MYO6*—we verified that the correct gene was not found among the model’s top 3 predictions. This outcome highlights how conflicting audioprofiles and clustering results can indicate that a prediction should be viewed with caution.

#### Case 3: *WFS1* Gene—Patient 3 (ID 13)

**Figure 4.**
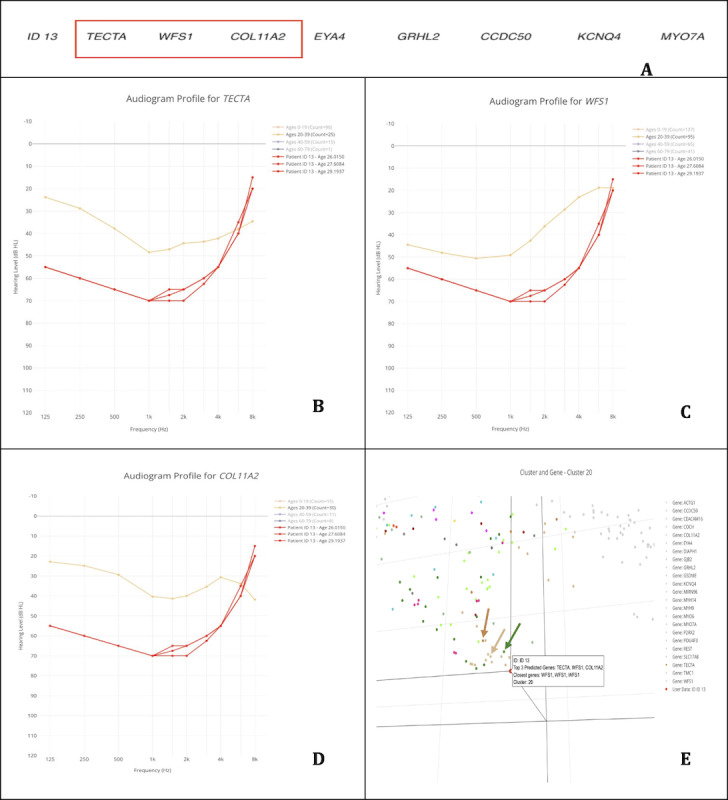
Application of the AudioGene Translational Dashboard for patient-level gene prediction, audiometric profile comparison, and cluster-based visualization. (A) Predictions for the patient (ID 13), with the top 3 genes being TECTA, WFS1, and COL11A2; (B) audioprofile of TECTA with the patient’s (ID 13) audiograms in red, taken at the ages of 26, 27, and 29 years; (C) audioprofile of WFS1 with the patient’s (ID 13) audiograms in red, taken at the ages of 26, 27, and 29 years; (D) audioprofile of COL11A2 with the patient’s (ID 13) audiograms in red, taken at the ages of 26, 27, and 29 years; and (E) 3D plot of audiograms in the training set converted into 3 dimensions, with genes in cluster 20 colored (genes not in cluster are light gray; patient [ID 13] is the red dot hovered over the displayed label; and the light brown arrow points to WFS1 [light brown dots], the green arrow points to MYO7A [green dots], and the brown arrow points to TECTA [brown dots]).

In our third case, the patient (ID 13) was diagnosed with *WFS1*-related hearing loss and, based on 3 audiograms, was predicted by AG4 to have *TECTA*-related, *WFS1*-related, or *COL11A2*-related hearing loss (Figure 4A).

When examining these predictions in relation to the patient’s audiograms, the following conclusions emerge regarding why *WFS1* is likely the correct gene and is captured within the top 3 predictions. First, the audioprofile for *TECTA* (Figure 4B) shows some similarities; however, it does not fully capture the nuanced relationship between low-frequency and high-frequency thresholds observed in the patient’s data. Second, *COL11A2* (Figure 4D) also exhibits differences that diverge from the patient’s pattern. Finally, *WFS1* (Figure 4C) demonstrates an especially close match to the patient’s audiometric profile, particularly in the way it mirrors better hearing at the high frequencies relative to the low frequencies. Although one might contend that *TECTA* or *COL11A2* could also be considered candidates based on partial pattern matches, the overall evidence—supported by the 3D clustering in Figure 4E—reinforces that *WFS1* provides the best fit.

Thus, whether one emphasizes the possibility of *TECTA* or *COL11A2* as contenders, the integrated data confirm that the correct gene, *WFS1*, is indeed within the top 3 predictions. This close alignment between the patient’s audiometric data and the *WFS1* reference profile, combined with supporting clustering analysis, enhances confidence in the diagnostic utility of the AudioGene Translational Dashboard.

## Discussion

### Principal Findings

These studies demonstrate how the model can raise or lower confidence in variant interpretation based on whether the correct genetic cause of ADNSHL appears among the top 3 predicted genes. Cases 1 and 3 illustrate scenarios in which the model successfully includes the causative gene in its top predictions and closely matches the patient’s audiometric data, thereby justifying a higher level of trust in the result. In contrast, case 2 underscores how mismatched audioprofiles and inconclusive clustering can reveal when the actual gene of interest is likely missing from the top 3 predictions. The interactive visualizations of the AudioGene Translational Dashboard, such as the APS and spatial analysis tools, remain valuable in identifying gene-specific patterns that align with clinical observations [20].

However, there are important limitations of the AudioGene Translational Dashboard, especially concerning smaller gene classes. The sparsity of data and the lower accuracy of models in these categories can make the AudioGene Translational Dashboard and phenotypic predictions less reliable. However, by presenting visualizations of the data distribution and class imbalance, these limitations become more apparent, allowing data interpretation to be adjusted accordingly [2,4].

### Conclusions

The AudioGene Translational Dashboard represents an advancement in the field of genetic diagnostics for ADNSHL. By integrating advanced ML algorithms with interactive visualization tools, the AudioGene Translational Dashboard enhances health care providers’ ability to interpret genetic data and make more informed diagnostic decisions.

A central feature of the AudioGene Translational Dashboard is the “70/30” phenomenon, which provides health care providers with critical context for confidence in genetic predictions. When the top 3 predictions are likely to contain the correct gene, the tool serves as a “green flag” for health care providers, increasing diagnostic confidence. Conversely, it alerts health care providers when predictions may be less reliable, serving as a “red flag” and prompting further investigation.

The AudioGene Translational Dashboard is an example of XAI in clinical settings, offering a context-driven method with increased transparency for the diagnosis of ADNSHL. Future developments will focus on incorporating custom model building, enhancing class imbalance functionality, and implementing user suggestions. The AudioGene Translational Dashboard not only advances genetic diagnostics for hearing loss but also serves as an example of a hybrid ML system.

## Abbreviations

ADNSHL: autosomal dominant nonsyndromic hearing loss
AG4: AudioGene version 4
AG9.1: AudioGene version 9.1
APS: audioprofile surface
KNN: k-nearest neighbor
ML: machine learning
SERN: SQL, Express.js, React.js, and Node.js
SVM: support vector machine
UMAP: uniform manifold approximation and projection
XAI: explainable artificial intelligence
